# On the Disintegration of A1050/Ni201 Explosively Welded Clads Induced by Long-Term Annealing

**DOI:** 10.3390/ma14112931

**Published:** 2021-05-29

**Authors:** Izabella Kwiecien, Anna Wierzbicka-Miernik, Maciej Szczerba, Piotr Bobrowski, Zygmunt Szulc, Joanna Wojewoda-Budka

**Affiliations:** 1 Institute of Metallurgy and Materials Science, Polish Academy of Sciences, 25 Reymonta St., 30-059 Cracow, Poland; a.wierzbicka@imim.pl (A.W.-M.); m.szczerba@imim.pl (M.S.); p.bobrowski@imim.pl (P.B.); j.wojewoda@imim.pl (J.W.-B.); 2 High Energy Technologies Works ‘Explomet’, 100H Oswiecimska St., 45-641 Opole, Poland; zygmunt.szulc@explomet.pl

**Keywords:** explosive welding, aluminum, nickel, interface, intermetallic phase

## Abstract

The paper presents the microstructure and phase composition of the interface zone formed in the explosive welding process between technically pure aluminum and nickel. Low and high detonation velocities of 2000 and 2800 m/s were applied to expose the differences of the welded zone directly after the joining as well as subsequent long-term annealing. The large amount of the melted areas was observed composed of a variety of Al-Ni type intermetallics; however, the morphology varied from nearly flat to wavy with increasing detonation velocity. The applied heat treatment at 500 °C has resulted in the formation of Al_3_Ni and Al_3_Ni_2_ layers, which in the first stages of growth preserved the initial interface morphology. Due to the large differences in Al and Ni diffusivities, the porosity formation occurred for both types of clads. Faster consumption of Al_3_Ni phase at the expense of the growing Al_3_Ni_2_ phase, characterized by strong crystallographic texture, has been observed only for the weld obtained at low detonation velocity. As a result of the extended annealing time, the disintegration of the bond occurred due to crack propagation located at the A1050/Al_3_Ni_2_ interface.

## 1. Introduction

The designing and manufacturing of metallic multilayered composites are one of the challenges of modern materials engineering. Difficulties associated with this may arise due to different chemical and physical properties of welded metals, the intermetallic phase formation at the interface, and the occurrence of defects such as voids or cracks within the bonded area [1,2,3]. Nevertheless, the metallic composites, because of their unique properties, may be potentially applied in many branches of industry, including automotive, aerospace, or military. Recently, explosive welding (EXW) has gained interest in being recognized as a successful method of multilayer materials manufacturing. Explosive welding may be utilized in a wide range of materials joining variants each characterized by different chemical properties, e.g., Al/steel [4], Al/Ti [5], Al/Cu [6], Al/Mg [7], or Al/Ni [8], Zr/steel [9], Ti/Ni [10] or Cu/Ti [11]. It is also suitable for bonding the same material such as Al/Al [12] or steel/steel [13]. Although the EXW is classified as a welding method [14], in contrast to classical welding, the presence of a heat-affected zone, which may have a destructive effect on the weld, is excluded [15]. On the other hand, in the case of clads manufactured by explosive welding, the phenomena such as local melting at the interface [5,7,8,16] and grain refinement of the parent material within the regions directly adjacent to weld may occur [17].

**Figure 1:**
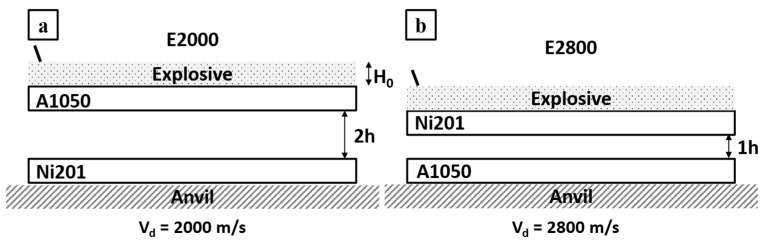
Scheme of the experimental set-up of (**a**) A1050/Ni201 manufactured with V_d_ = 2000 m/s and (**b**) Ni201/A1050 manufactured with V_d_ = 2800 m/s.

The explosive welding set-up usually consists of two parallel plates separated by woodblocks defining the distance between them and an explosive mixture with igniter on the top of the upper plate (Figure 1) [5,17,18,19,20,21]. The EXW process may also take place using different set-up, e.g., in inclined configurations [20] or under water [22,23]. Energy derived from the explosion causes acceleration of the top plate, called a flyer plate (fp), toward the base plate (bp) localized on the ground, which collide at a specific angle (β) and impact velocity (V_p_), forming the bond at the interface between the plates. The interface formation depends on many factors, i.e., type of joined metals [7,10,24,25]; explosive ratio (R), which is associated with the amount and type of the explosive mixture [24,25,26]; initial distance between the colliding plates called stand-off distance (SOD) [9,19,27,28]; and mutual localization of the colliding plates [7,8,27]. The surface cleaning is supported by the jetting effect during explosive welding, resulting from the high pressure at the collision point from which the jet is ejected outside. The jet action removes the outer layer of the surface with all its impurities [29]. Importantly, the explosive welding technique is sensitive to any changes of the bonding parameters mentioned above. Minor differences may affect the interface morphology, microstructure, and mechanical properties [24,25,27,30]. 

Aluminum and nickel are an interesting joining couple for several reasons. First of all, the high quality of the bond between these metals is difficult to obtain due to the large difference in their melting temperature (1483 °C versus 660 °C). Moreover, according to the equilibrium diagram [31], the intermetallic phase formation may occur at the interface [8,32]. Aluminum and nickel interaction was intensively studied at conditions close to the equilibrium state—diffusion couple experiment [33] or diffusion soldering process [34,35,36], which may serve as a starting point for further consideration of non-equilibrium joining. Other ways of Al/Ni multilayers formation involve accumulative roll bonding [37], magnetron sputtering process [38], or casting [39]. However, these methods are associated with insufficient strength of bond or constraints related to the size of the manufactured item. Despite the large potential application, the explosive welding of the Al/Ni system is still needed to be fully explored. The first possibility of joining Ni with Al by EXW was presented by Gerland et al. [3], who manufactured thin bimetallic plates. Further important experiments involving the production of the multilayer were described by Bataev et al. [8,32], where the morphology differences of Al/Ni multilayered composites dependent on the localization of the interface—the bond was either flat (Ni/Al interface) or irregular with broader continuous melted regions (Al/Ni interface), were emphasized. It has also been shown that this may not be a general rule particularly in the case of low detonation velocity [21]. Thus, it is essential to conduct in-depth experiments at different explosive welding parameters (e.g., extremely high or low detonation velocity) to obtain a comprehensive description of the materials properties. On the other hand, the influence of SOD on the microstructure and mechanical properties of Ni/Al/Ni three-layered composites were studied by Guo et al. [27], where the bonding interface changed from near flat to wavy. Authors argued that for the multilayered systems, the stand-off distance is the most important technological parameter, which determines the final bond quality. In these papers, the heat treatment effect applied after EXW was only briefly studied. [3,8,27,32]. Gerland et al. [3] and Guo et al. [27] have not studied the heat treatment influence on the explosively welded Al/Ni clads. On the other hand, Bataev et al. [8] and Ogneva et al. [32] indicated that only Al_3_Ni and Al_3_Ni_2_ phases formed after annealing at 620 °C for the relatively short annealing time ranging from 5 min to 5 h. In both these papers, the Al/Ni multilayered composites subjected to the annealing revealed faster growth of the intermetallic layer in EXW clads in comparison to the cast samples. Bataev et al. [8] explained it by a presence of the clean surfaces without oxides or impurities after the joining process. Moreover, the annealing led to transformation of the metastable phases or decagonal phases to the stable phases. According to the images in [32] presenting the Al/Ni interfaces after annealing, two types of the interface shape were formed, preserving the morphology observed directly after explosive welding. After 5 h of annealing at the boundary between Al_3_Ni and Al_3_Ni_2_, authors of [32] noted the presence of a porous layer; however, they did not comment on it in detail. The heat treatment is an important technological step, necessary to be applied for internal stress relaxation or in formation of metallic-intermetallic laminates [5]. Current work exposes the influence of detonation velocity, arrangement of the plates and their stand-off distance on the microstructure and phase formation. Additionally, the post-processing heat treatment was applied to demonstrate its influence on the interface zone transformation and the resulting reliability of the bond. Presented studies are essential due to the sensitivity of the Al-Ni system on the occurrence of the Kirkendall effect, especially at the conditions of low temperature annealing, where substantial inequality of atomic movement between the joined alloys occurs. So far, the effect of the joining conditions on the microstructural transformation after long-term annealing and phenomena accompanying them in the case of explosively welded Al/Ni interface has not been studied.

## 2. Materials and Methods

**Figure 2:**
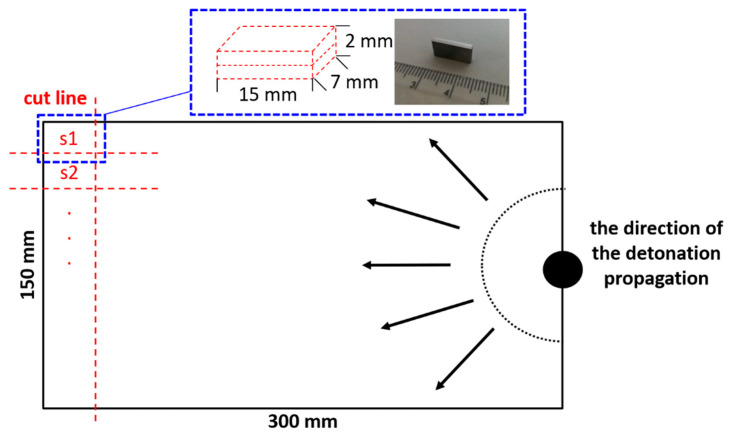
Schematic representation of sample cutting with respect to the direction of the detonation propagation.

The A1050 aluminum alloy Alcomet (Szumen, Bułgaria) (herein called Al) was joined by explosive welding with the Ni201 nickel alloy Nippon Yakin Kogyo Co., Ltd., International Plaza, Singapur (herein called Ni). Chemical compositions of the flyer and base plates are collected in Table 1. As it is shown in scheme of the experimental procedure (Figure 1), both bimetallic plates were manufactured in the parallel system using two sets of process parameters. To study extreme joining conditions, EXW clads were manufactured with the application of entirely various detonation velocities. Explosive welding set-up prepared to manufacture Ni/Al clad with lower detonation velocity (V_d_) of 2000 m/s was composed of Al acting as a flyer plate accelerated toward the Ni base plate located with the stand-off distance denoted as 2h (Figure 1a). In the second case, represented in Figure 1b, the applied detonation velocity of 2800 m/s was exceptionally high [40]. In such case, the nickel alloy was applied as a flyer plate and aluminum alloy as a base plate with a shorter distance between the plates of 1h (Figure 1b). The EXW clads manufactured with low and high detonation velocity will be called E2000 and E2800, respectively. The explosive welding parameters are collected in Table 2 and presented in Figure 1. After explosive welding, smaller samples (15 × 7 × 2 mm^3^) were cut out for microstructure observations (Figure 2). Additionally, part of these samples were annealed at 500 °C for different time intervals (0.5–168 h). The cross-sections of the samples for further electron microscopy observations before and after annealing were prepared using standard metallographic procedures.

Scanning Electron Microscope (SEM) FEI Quanta FEG (FEI, Hillsboro, OR, USA) equipped with EDAX Apollo Energy Dispersive X-ray Spectrometer (EDS) was used for the microstructural observation at the micro-scale and the chemical composition analysis at the interface zone. Further microstructure analysis was performed by electron backscattered diffraction (EBSD) technique using EDAX Hikari device. The phase analysis for the explosive welded sample after annealing at 500 °C for 168 h was performed by X-ray diffraction technique (XRD) using Bruker D8 diffractometer (Bruker, MA, USA) with Co radiation. The measurements were performed in the diffraction range from 20 to 140 degrees of 2θ. The differential scanning calorimeter (DSC 404 F1 Pegasus, Netzsch, Selb, Germany) was used for temperature selection of annealing based on the A1050 alloy melting temperature. The experiment was performed in the pure argon protective atmosphere with the heating and cooling rate of 10 K/min in the temperature range of RT-700 °C.

## 3. Results and Discussion

### 3.1. Microstructural Characterization after Welding

**Figure 3:**
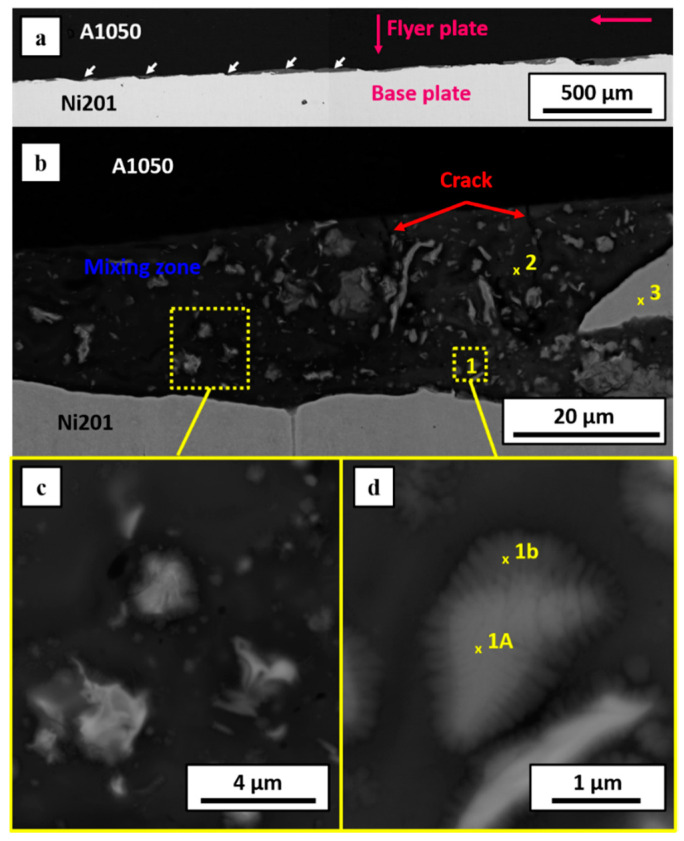
Microstructures of explosively welded A1050/Ni201 clad manufactured at V_d_ = 2000 m/s: overall view of wavy interface (**a**), representative part of the melted region of individual wave with its microstructure imaged by backscattered electrons contrast in SEM (**b**) together with the magnification of the microstructure details (**c**,**d**). Results of chemical analysis marked with points: 1A, 1b, 2 and 3 are collected in Table 3.

**Figure 4:**
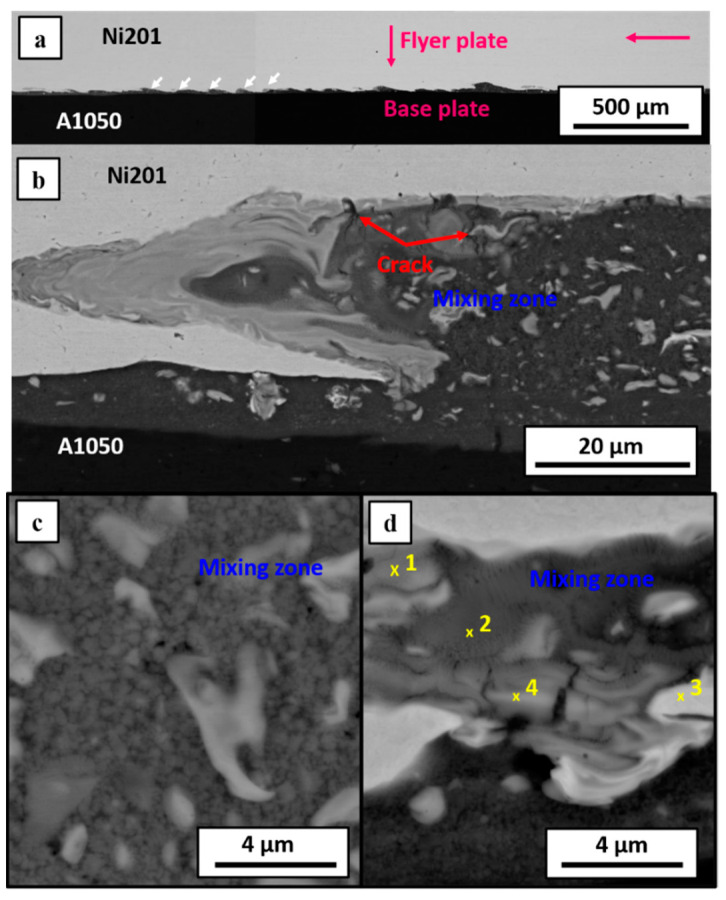
Microstructures of explosively welded Ni201/A1050 clad manufactured at V_d_ = 2800 m/s: overall view of wavy interface (**a**), representative part of the melted region of individual wave with its microstructure imaged by backscattered electrons contrast in SEM (**b**) together with the magnification of the microstructure details (**c**,**d**). Results of chemical analysis marked with points 1 to 4 are collected in Table 4.

The applied detonation velocity of 2000 m/s was high enough to obtain continuous weld between Al and Ni plates (Figure 3a), the same as in the case of very high detonation velocity of 2800 m/s, reported previously in [40]. In both these cases, the asymmetric waves at the interface are observed. Similar observations were made by Fronczek et al. [41] studying Ti/Al, Shiran et al. [42] for steel/Al, or by Carvalho et al. [43] in the case of explosively welded Cu/Al clads. The explanation of such waves shape is associated with the significant difference in the melting temperature and density of the joined plates. In the case of aluminum and nickel, the melting point and density differences of both metals reach almost 800 °C and 6.20 g/cm^3^, respectively. When the density of the colliding plates is similar, the waves occurring at the interface should be symmetric [44]. Nevertheless, the overall appearance of the welded zone in the studied case is different. First of all, the average height and distance between the waves were determined to be as follows: 20 µm, 160 µm and 30 µm, 80 µm, respectively for E2000 and E2800. The thickness of the interface was defined as the average height of waves measured as a distance between the highest point at the crest of the wave to the base line indicating the place where the wave began to form. The interface of E2000 presented in Figure 3a consists of melted areas along the whole length of the bonding zone, and it is flatter in comparison to the E2800 (Figure 4 in current work and Figure 2a in [40]), where the obvious wavy morphology prevailed. Such difference in the appearance of interfaces may result from few factors. First of all, it may be associated with the applied and significantly different detonation velocity. Loureiro et al. [26], who investigated the effect of explosive ratio (R) on the quality of Al/Cu interface, indicated that the increase of R parameter leads to the increasing detonation (V_d_) and impact (V_p_) velocities and results in a slight change of collision angle β. By increasing the explosive ratio and thereby the detonation velocity, they caused the increase of the amplitude of the waves and the appearance of Cu islands inside the aluminum plate due to the peel-off of small parts from the cooper plate [26]. In the work presented by Paul et al., parts of the parent materials of Cu and Ti were also observed, but in that case, they were enclosed within the melted region [11]. In the current work, authors also noted the Ni islands in both of the studied bimetallic plates. In the case of the lower detonation velocity (E2000), they occurred more frequently, which is opposite to results presented by Loureiro et al. [26], where the occurrence of Cu islands was related to higher detonation velocity. As there seems to be no consistent tendency of metallic islands formation versus the applied detonation velocity, it may be assumed that this is an individual feature of the specific explosive weld. Generally, the higher detonation velocity should favor the formation of such phenomenon [29]. Thus, the relation shown here may also be associated with the applied material of the flyer plate. The literature suggests that the metal of lower density and tensile yield strength should be used as a flyer plate [45]. Contrary to this suggestion, in the case of E2800, nickel alloy was accelerated toward the aluminum. Worthy attention is the localization of the Ni particles being embedded rather within the melted zones than within the aluminum plates. As in our previous study [21], it was shown that the mutual localization of aluminum and nickel alloys plates colliding at the 2400 m/s detonation velocity does not significantly influence the overall appearance of the weld. It can be presumed that the difference observed here may result from the simultaneous effect of several factors. Besides the significantly reduced detonation speed (2000 contrary to 2800 m/s), causing the formation of a nearly flat melted interface, the effect of the stand-off distance should be considered, which in the case of E2000 was significantly higher than for E2800. The slight difference in terms of the wave morphology and grain refinement occurring in [21] was also assigned to the SOD. Generally, there is a tendency to broaden the bonding zone with increasing SOD [27]. Paul et al. [11] have shown a series of microstructure images of Ti/Cu explosive welds, manufactured with the application of different V_d_ in the range of 2000–3000 m/s and SOD increasing gradually by 1.5 mm in the range of 1.5–9.0 mm, where this relationship can be directly followed. Results presented here and in [40] show that the increase of V_d_ and SOD causes an increase of waves’ amplitude and distance between their crests as in [11]. Guo et al. [27] studied Ni/Al/Ni sandwiches manufactured by EXW with the detonation velocity of about 2100 m/s, indicating that the melted regions are more pronounced when the SOD is higher. Three different kinds of the interface were obtained: straight, wavy, or continuously melted, changing with the increasing SOD [27]. 

Detailed microstructural observations carried out in this study for both E2000 and E2800 (Figure 3b–d and Figure 4a–d) revealed typical rapidly solidified microstructure, particularly expressed by fine dendritic microstructure with visible core and arms. As it is presented in [40], in the case of E2800, the average size of primary arms of the dendrites was about 480 nm (range of 250 to 670 nm), and secondary arms reached an average length of 240 nm (range of 150 to 350 nm). Reduction of detonation velocity to 2000 m/s resulted in the presence of dendrites with the mean size of primary arms of about 280 nm (being in the range of 230 to 400 nm), while the precise determination of secondary arms size was impossible based on the SEM images. The distribution of the dendritic microstructure is different in both cases of explosive clads. In the case of the clads obtained using lower detonation velocity, the dendrites were mainly observed in the surroundings of the other microstructural elements and rarely occurred within the melted zone freely. On the other hand, the dendritic microstructure constituted the matrix for precipitations within the interface of the E2800 clad. Moreover, the size of the dendrites was related to the localization within the melted region, and it can be observed that the free or matrix dendrites were smaller than the dendrites bonded with precipitations or brittle crests of the waves (Figure 3d and Figure 4c) and their size is related to the cooling rate. 

Melted regions presented in Figure 4c,d are similar to those observed by Bataev’s group and described in [8,32]. In our case, the melted zone is inhomogeneous and consists of the areas of different SEM-BSE contrast. The common feature of both kinds of the weld is the crack presence across the melted zone, which is justified by the presence of the brittle intermetallic phases and shrinkage of materials due to the fast-cooling rate and solidification. The sharp increase of the temperature during EXW exceeds the transfer of the generated heat outside the collision point. As a result of non-effective heat dissipation, local melting with intensive mixing of the chemical elements at the interface occurs. The cooling at the collision place becomes more effective as the detonation front proceeds [46,47], and the extremely high cooling rates promote the cracking process. Additionally, besides dendritic structures, larger particles of brighter contrast exist. The SEM-EDS chemical composition analyses were conducted from the representative places of the mixing zone. The results were collected in Table 3 and Table 4, where the points correspond to the locations marked in Figure 3b,d, and Figure 4b, respectively. These EDS measurements allowed to determine the chemical composition of the intermetallic phases from Al-Ni equilibrium phase diagram [31]. The presence of phases such as Al_3_Ni, Al_3_Ni_2_ and AlNi was confirmed. These results are consistent with our previous studies [21,40], where additionally numerous TEM-EDS analysis were conducted. Application of TEM technique was necessary due to the high inhomogeneity of the investigated areas in [40], their refinement and insufficient analytical SEM/EDS resolution for this study. This allowed to observe the additional metastable Al_9_Ni_2_ phase in the E2800 clad case [40], which was also found in the case of Al/Ni multilayered composites manufactured with extraordinary high detonation velocity in [8].

**Figure 5:**
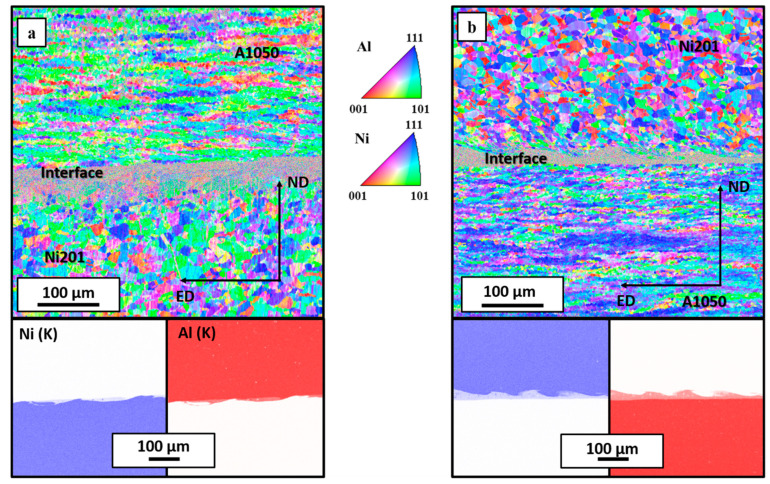
EBSD maps and EDS elemental distribution maps of Al (K) and Ni (K) of the explosively welded clads’ interfaces and adjacent regions obtained using 2000 m/s (**a**) and 2800 m/s detonation velocity (**b**).

Grain refinement of the interface region can be compared by analyzing two examples of EBSD and EDS elemental distribution maps collected from large regions of both clads (Figure 5). Due to the high chemical composition inhomogeneity of the melted area [41], strong deformation of material near the interface [48] and grains’ refinement [17], proper indexing of these areas is quite challenging. The interface regions constitute randomly oriented fine grains. Nevertheless, the presented results exclude the crystallographic texture of Al and Ni alloys in both EXW clads. The microstructure of nickel alloy indicates it is mainly built from equiaxed grains with an average size of 9.1 and 7.2 μm for E2000 and E2800, respectively. Similar results were presented in [21], where depending on the mutual localization of the colliding plates, the final size of Ni grains varied substantially. In [21], where the detonation velocity of 2400 m/s was used and the main variable was the mutual localization of the colliding plates when Al alloy acted as a flyer plate, the grains of Ni alloy were more refined in comparison to the clad when Ni was used as a flyer plate. When aluminum was used as a flyer plate, the distance between the plates was doubled in comparison to Ni/Al EXW clad, similar to in current work. Moreover, the grain refinement was correlated with the thermal conductivity of Al and Ni alloys, and therefore, the heat transfer from bimetallic plates after the EXW process is different. Both detonation velocity and stand-off distance influence the nickel grain size. In [21], it was shown that with an increase of SOD, the grain size of nickel decreases. Additionally, the Ni mean grain size also decreases at higher V_d_. When comparing the nickel grain size for E2000 with Al/Ni weld reported in [21], manufactured with the same SOD but with higher V_d_, the effect of V_d_ on the Ni grain size was confirmed. The same relationship holds comparing E2800 EXW clad and Ni/Al presented in [21], where also SOD was consistent, and only V_d_ differed. In conclusion, it may be assumed that both V_d_ and SOD influence the Ni grain refinement after EXW. Thus, higher SOD in the case of the E2000 clad reduces the effect of significantly higher V_d_ applied for E2800. 

### 3.2. Microstructure Characterization after Welds’ Annealing

**Figure 6:**
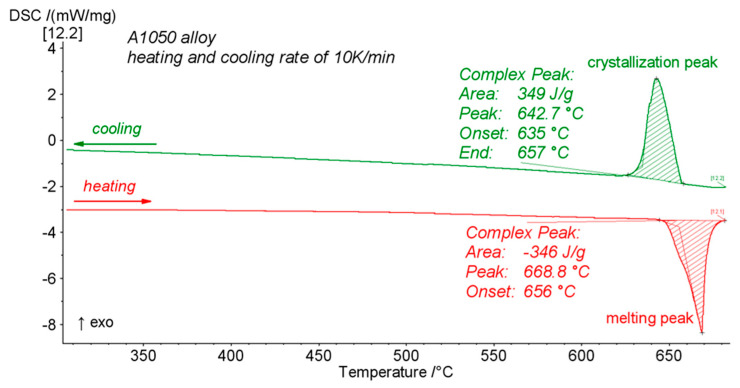
DSC curve registered for A1050 plate with the heating rate of 10 K/min.

Controlling the growth of the intermetallic phase during annealing is extremely important not only due to further possible applications [49,50] but, as it will be shown, also to predict (and avoid) serious delamination failure. Both types of EXW clads were annealed at 500 °C for a different time ranging from 0.5 to 168 h. The selection of the annealing temperature was based on the melting temperature of A1050 alloy experimentally determined at 656 °C, where melting begins (Figure 6). This allowed the aluminum alloy melting to be avoided and to conduct the observations of the microstructural changes taking place in the solid state. 

**Figure 7:**
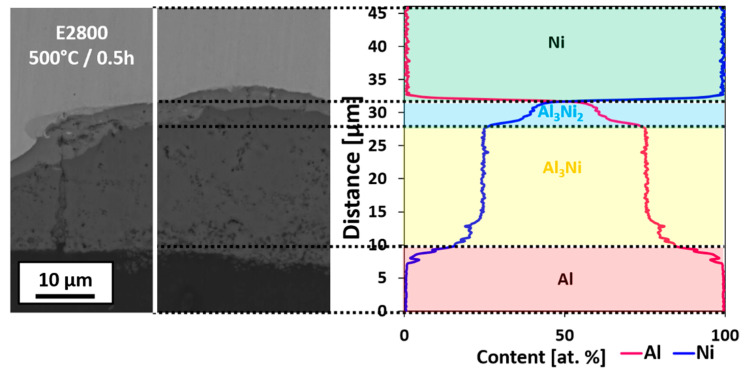
SEM image of the cross-sectional view of the interface zone in Ni201/A1050 manufactured at V_d_ = 2800 m/s after 0.5 h of annealing at 500 °C with the EDS line-scan across the interface area.

**Figure 8:**
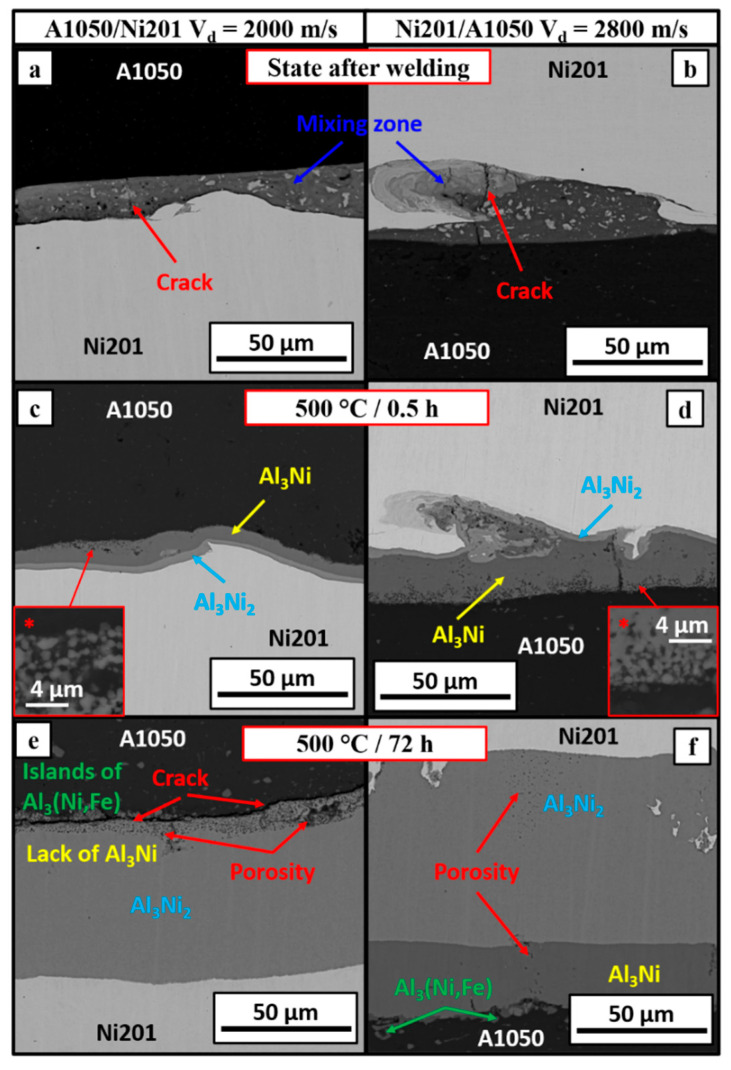
Comparison of the microstructure of the interface zone in A1050/Ni201 and Ni201/A1050 clads in the state after explosive welding (**a**,**b**) and after annealing at 500 °C for 0.5 h (**c**,**d**) and 72 h (**e**,**f**). The clads were manufactured at 2000 m/s (**a**,**c**,**e**) and 2800 m/s (**b**,**d**,**f**) detonation velocity.

Chemical composition analysis revealed that the interfaces of short time heat-treated EXW samples were composed of Al_3_Ni and Al_3_Ni_2_ phases located in the following order: A1050/Al_3_Ni/Al_3_Ni_2_/Ni201. This sequence of phases holds for both types of explosively welded clads (please compare to [40]). Further prolongation of annealing time led to creating the third compound at the boundary between Al_3_Ni and A1050. This phase was additionally enriched in iron, being an additive of both initial alloy plates (Table 1). Figure 7 shows the change of the chemical composition across the melted region. Some chemical composition fluctuations occurred within the Al_3_Ni_2_ phase, most probably due to the small thickness of this phase and collection of the X-rays from the adjacent region of the Al_3_Ni phase and Ni alloy. Fluctuations of the chemical composition in the neighborhood of Al_3_Ni/Al alloy interface results from the morphology of newly growing Al_3_Ni. The small grains grew within the aluminum matrix (as it was also observed in [51] and therefore at the earlier stage of the growth, the presence of some not-reacted Al regions between newly formed Al_3_Ni grains causes the fluctuations in chemical composition across the line-scan. This effect will be analyzed in more detail later on in the text. The composition of the phases has been assigned based on the equilibrium phase diagram described in [31]. Of course, even long-term annealing does not guarantee that the equilibrium state is attained. However, the identification of phases based on the equilibrium phase diagram in this process is much more justified than in the case of the initial interface created after explosive welding, where high pressure, local melting and fast crystallization occur. According to the equilibrium phase diagram, phases such as Al_3_Ni compound, consisting of 75 at. % of Al and 25 at. % of Ni and Al_3_Ni_2_ existing in the range of 59–63 at. % of Al and 37–41 at. % of Ni, can be found. Al_3_Ni phase has an orthorhombic structure with Pnma space group [52], while the Al_3_Ni_2_ phase is of a rhombohedral structure with P-3m1 space group [53]. The sequence of the intermetallic phase formation in Al-Ni system has been a subject of many studies [35,36,54,55]. Based on them, it is difficult to define the sequence of phases formation clearly. Some of the works indicate that the initial forming phase is Al_3_Ni followed by Al_3_Ni_2_ [34,35,36]. On the other hand, some modeling results or in situ experiments indicate that the formation of Al_3_Ni is preceded by Al_3_Ni_2_ formation [54,55]. Nevertheless, incompatibility in terms of the sequence of the intermetallic formation is not the case for explosive welding clads, at least for the first stages, because the initial interface has been already formed in extreme conditions of temperature and pressure before bimetallic plates were annealed. As a result of a metastable process, which is a consequence of the explosion and collision of reacting plates with high speed, the interface consisting of a mixture of different phases is formed. Only half an hour of annealing results in creation of both Al_3_Ni and Al_3_Ni_2_ phases in the form of continuous layers (Figure 8c,d). Both these phases grow simultaneously from the first stage of their formation. Previous studies have shown [56] that in the case of the Al/Ni diffusion couple, the occurrence of the intermetallics at the interface is correlated to the temperature of annealing. One hour of annealing at 450 °C resulted in the formation of the Al_3_Ni phase only, while at 500 °C, both Al_3_Ni and Al_3_Ni_2_ were observed. At higher temperatures after a short period of annealing, both Al_3_Ni and Al_3_N_2_ intermetallic phases coexisted as was described in [57,58]. Contrary to experiments performed in the solid-state, during the diffusion soldering experiment at 720 °C after 30 min of annealing, Al_3_Ni_2_ AlNi and AlNi_3_ phases were identified [34,59].

**Figure 9:**
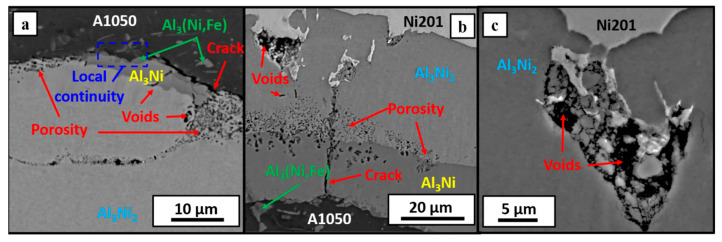
Defects of the welds occurring at the interface after annealing at 500 °C for 24h for the explosive welds obtained at 2000 m/s (**a**), 2800 m/s (**b**,**c**) detonation velocity.

The sequence of the microstructure changes at the interfaces of both types of clads after explosive welding and subjected to heating at 500 °C for various time are presented in Figure 8. Figure 8a,b presents the initial microstructure of both explosive welds. The significant differences may be observed already after explosive welding—the melted layer, which was formed in the case of low detonation velocity, is continuous and nearly planar, keeping almost constant thickness at the whole length. In comparison to E2800 (Figure 4a), there were only exceptionally local variations in the form of less often distributed waves of low amplitude showed in Figure 3a. The overall more complex appearance of the clad interface obtained with higher detonation velocity was in detail described in [40], and for the comparison, it is also presented in Figure 4. It consists of alternating waves with solidified melted regions. The morphology of the intermetallic layers in the first stage of growth is a replica of the interface zone morphology after explosive welding. As it is shown in Figure 8c,d, after short-term annealing, some microstructure features of the primary interface were preserved, and it is still possible to distinguish a wavy character. The Al_3_Ni phase formed as a continuous layer adjacent to the A1050 alloy is characterized by different thicknesses (E2000—7.7 µm versus E2800—16.1 µm), being broader in the case of clad obtained under higher detonation velocity. The second phase—Al_3_Ni_2_ (thickness of about 2.5 µm) after 0.5 h of annealing exists in the form of thinner than Al_3_Ni layer located near the nickel alloy. Moreover, at the melted regions within the waves present in the E2800 clad, the areas of mixing of both Al_3_Ni and Al_3_Ni_2_ phases can be observed. It is important to notice the peculiar microstructure of the initially growing Al_3_Ni phase presented in Figure 8c *,d *. The small, nearly equiaxed grains, densely distributed, are growing and finally joining with each other and therefore forming the continuous Al_3_Ni layer (Figure 8f). Such growth character was previously observed and described in other joining process [60], where the addition of the Ni into Cu caused an acceleration of the intermetallic growth after reaction with Sn. The phase growth in the form of the nodes was also observed by Fronczek et al. in other explosively welded system (TiAl_3_ phase in Al/Ti) [51]. Longer time of annealing revealed even more significant differences between the studied interfaces. Prolonged annealing of the EXW clads joined with low detonation velocity of 2000 m/s resulted in consumption of the Al_3_Ni by the growing Al_3_Ni_2_ phase, which residuals still locally existed after 24 h (Figure 9a) and not have been detected after longer time of 72 h of annealing (Figure 8e). Contrary to this, SEM observations for clads obtained using high detonation velocity (2800 m/s) evidenced the presence of the Al_3_Ni of about 25 µm in thickness at the interface after 72h of annealing. Independently of the applied detonation velocity, the longer annealing time, the broader the thickness of the Al_3_Ni_2_ phase (being of about 55 µm for E2000 and 75 µm for E2800 after 72 h). At the early stages of the growth, the thickness of Al_3_Ni phase was lower in the case of E2000, and prolongation of annealing time exposed even more the differences between both kinds of clads. In the case of the Al_3_Ni layer of the clad obtained using high detonation velocity, the thickness of the phase gradually expanded with time up to 72 h of annealing, achieving the maximum value and then started to decrease due to the growth of Al_3_Ni_2_ phase at its expense. On the other hand, in the case of EXW obtained using low detonation velocity, the Al_3_Ni phase did not broaden, and its thickness decreased already after further annealing, leading to complete disappearance after 72 h of annealing. In contrast to this behavior, the growth of the Al_3_Ni_2_ phase was similar for both explosively welded types of clads. At the beginning of the annealing (up to 24 h), the difference in thickness was within the measurement deviation; however, longer annealing showed a thicker layer in the case of E2800. After 72 h of annealing, the growth of Al_3_Ni_2_ was much slower or even completely inhibited due to the accumulated porosity occurring along the A1050/Al_3_Ni_2_ interface (Figure 8e), while in the case of E2800, further broadening of the Al_3_Ni_2_ phase was continued [40].

After the heat treatment inside of Al_3_Ni_2_ layer and on its boundary with nickel alloy, the brittle regions were observed (Figure 9c), and their presence results from the characteristic intermetallic phase growth (Figure 8d). The appearance of such regions involves the situation when some amount of Ni alloy is fully or partially enclosed within the Al_3_Ni_2_ phase like it is showed in Figure 8f. Annealing of these areas leads to the diffusion between Ni islands and their surroundings and ‘closing’ Ni islands by formation the small isolations combining with each other, the way it was observed in the case of initial growth of the Al_3_Ni phase. The phenomenon described above concerns only E2800 clad, as in the case of E2000 clad, lack of the brittle regions was recognized, despite the presence of the Ni islands. Therefore, apparently, the flatness of the interface may be presumed to be beneficial due to the reduction in a number of obstacles in the form of large areas of Ni201 alloy between melted waves and reduced time necessary to form continuous layers of intermetallic phases. The characteristic porosity is present in both types of clads. 

The SEM-BSE images of the E2000 clad interfaces in Figure 8 show that the porosity is located inside the Al_3_Ni_2_ phase in the neighborhood of aluminum alloy. Even if some small amount of Al_3_Ni phase still remained after annealing (shorter time), the porosity is located inside the Al_3_Ni_2_ next to A1050 alloy, while the Al_3_Ni_2_/Al_3_Ni boundary is locally continuous without porous regions (Figure 9a). When both of the phases simultaneously exist, the porosity is localized at their boundary (Figure 9b)—this situation takes place in E2800 clad. Additionally, some fraction of the porosity inside the clad is distributed linearly and perpendicularly to the phases interface within both intermetallic layers. The consequence of such intensive porosity distribution may be cracking at the Al_3_Ni_2_/A1050 interface without application of any external forces, which was observed in the case of E2000 annealed for 168 h (Figure 8e and Figure 9a). As it is presented in Figure 8e, the crack is localized at the phase boundary and locally passes through the Al_3_Ni_2_ phase; however, this defect is continuous at the whole length of the weld. This description refers to the situation of the complete lack of the Al_3_Ni phase. On the other hand, when some amount of this intermetallic phase is present after a shorter annealing time (Figure 9a), the areas of local continuity of bonding remained. 

Guo et al. [27] indicated the importance of the morphology of the interfaces in the state after welding. They reported that clads with straight and wavy interfaces have better tensile strength and elongation than the clads characterized by the interfaces of the continuous melted region. In the case of explosive welds presented in this study, the continuous melted layer was always observed; however, the overall form of both interfaces was different. Most likely, it has a crucial influence on the welds’ durability after annealing and is associated with the growth rate of the intermetallic phases. The presence of porosity within the interface zone may be related to two factors: (i) characteristic initial growth of Al_3_Ni and Al_3_Ni_2_ phases, which form as small isolations and subsequently combine with each other while remaining the spaces between them and (ii) with Kirkendall effect, which was demonstrated by Janssen [58] in 1967 during the experiment with Al/Ni diffusion couple. Authors indicated that the Kirkendall effect in the Al-Ni system is pronounced, and during the formation of Al_3_Ni and Al_3_Ni_2_ phases at 600 °C, only Al atoms are active. The growth of the other intermetallic phases is related to the movement of Ni atoms at higher temperatures.

**Figure 10:**
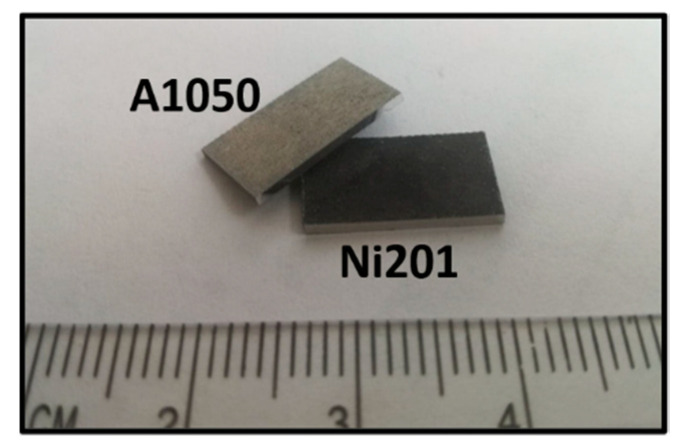
Disintegration of the Ni201/A1050 explosively welded clad manufactured by V_d_ = 2000 m/s after 168 h of annealing at 500 °C.

Further annealing of the clads obtained with lower detonation velocity resulted in the disintegration of the sample directly after its cooling with the furnace (Figure 10), while for the clad obtained at higher detonation velocity, the broadening of the interface due to the further growth of the intermetallics’ layers was continued.

**Figure 11:**
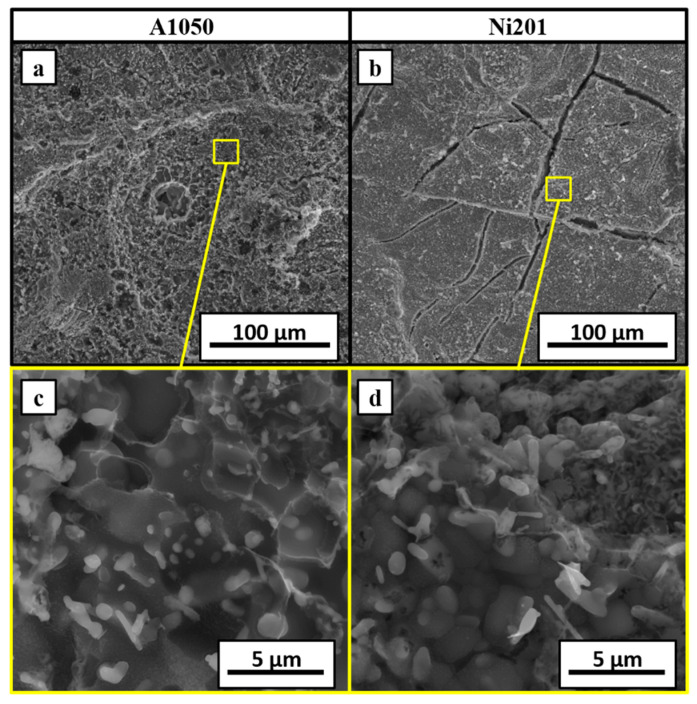
SEM-SE images of the surfaces’ fractures of A1050 (**a**,**c**) and Ni201 (**b**,**d**) side of EXW Ni201/A1050 clad manufactured at V_d_ = 2000 m/s and annealed at 500 °C for 168 h after disintegration.

**Figure 12:**
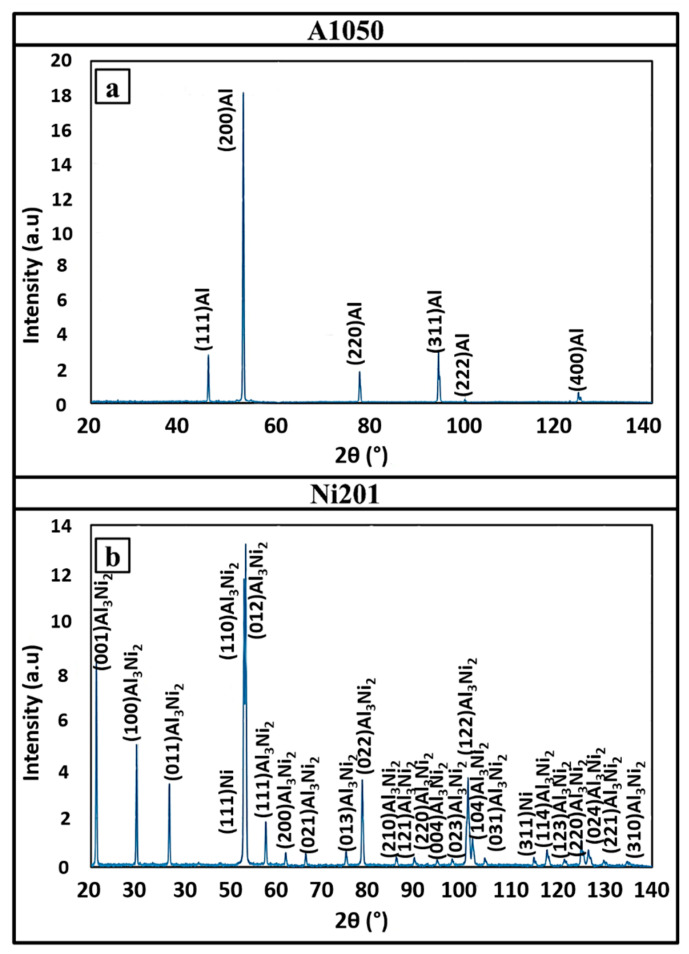
XRD patterns taken from the fracture surfaces of A1050 (**a**) and Ni201 (**b**) after the disintegration of Ni201/A1050 clad manufactured at V_d_ = 2000 m/s followed by annealing at 500 °C for 168 h after disintegration.

The SEM-SE images of both fractured surfaces are presented in Figure 11. The A1050 side (Figure 11a,c) is irregular and consists of many small shallow dimples. On the other hand, the second part (Ni201 side) is flatter; however, at the surface, many long and branched cracks can be noticed. XRD spectra obtained from both fracture surfaces let to determine their phases’ composition (Figure 12). The spectrum collected from the aluminum alloy side showed only the peaks of pure aluminum, while the one collected from the second side revealed the peaks coming mainly from Al_3_Ni_2_ phase and an additional few from the nickel at 2 theta of 53° and 114°—corresponding to the planes of (111) and (311), respectively (Figure 12b).

**Figure 13:**
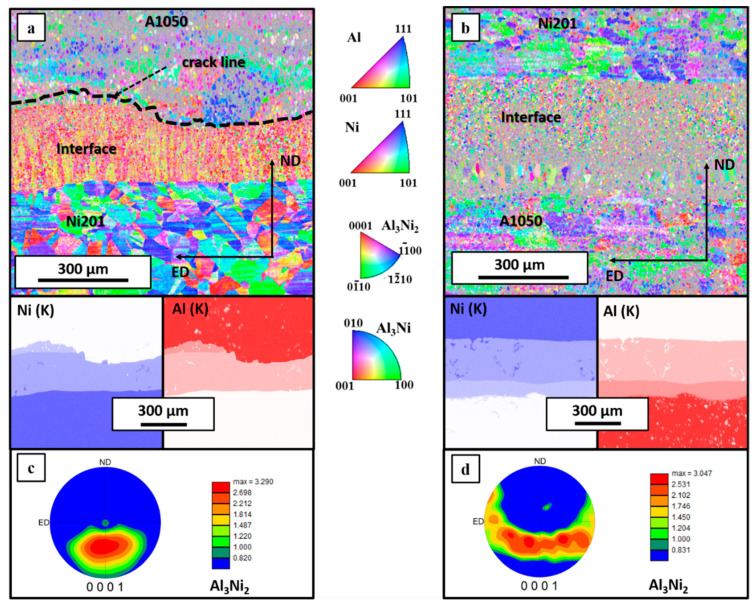
EBSD maps and EDS elemental distribution maps of Al(K) and Ni(K) corresponding to the interface zone after annealing at 500 °C for 168 h in E2000 (**a**) and E 2800 (**b**) explosively welded clads with pole figure generated for Al_3_Ni_2_ phase presented in (**c**) and (**d**), respectively.

Additional important information on the microstructure of the welds and therefore possible explanation of disintegration phenomenon can be found with the aim of electron backscattered diffraction technique. The EBSD maps collected from both EXW clads are presented in Figure 13a,b with the corresponding EDS elemental distribution maps confirming homogeneity of the chemical composition measured in the cross-sectional view of the connections. The EBSD maps allowed to reveal the ultrafine microstructure of the Al_3_Ni_2_ phase in both types of welds. Such fine grains were observed previously for A1050/Ni201 clads manufactured with a detonation velocity of 2400 m/s and were in detail described in [21]. Black dashed line marked along with the aluminum alloy and the interface zone (Figure 13a) represents the localization of the crack that occurred after annealing at 500 °C for 168 h. It should be noted that the grains of the Al_3_Ni phase in E2800 weld after long-term annealing are visibly larger than Al_3_Ni_2_ ones and are adjacent to A1050 alloy (Figure 13b). However, the most striking observation is the strong texture of the Al_3_Ni_2_ phase present only in the case of the Al_3_Ni_2_ phase in the weld obtained with lower detonation velocity (Figure 13c). Additionally, these textured small equiaxed grains are arranged in columns in the longitudinal direction perpendicular to the interface. Contrary to this, there is no texture in the case of the Al_3_Ni_2_ phase present in the weld obtained using higher detonation velocity (Figure 13d). The dominating orientation was localized near to the directions of <0001> and <1100> parallel to ND in the case of E2800, while for the sample produced at low detonation velocity, the dominating orientation was determined as near to the direction of <0001> parallel to ND. The pole figures collected for the Al_3_Ni_2_ phase revealed that for the E2000 weld the maximum was concentrated in the center of the lower part of the figure (Figure 13c) and for E2800 is slightly blurred (Figure 13d). As it has been already mentioned, aluminum is the more mobile element of the Al_3_Ni_2_ phase. Therefore, a very pronounced Kirkendall plane is located after annealing in the vicinity of the A1050/product intermetallic interface. Whether diffusional porosity (voiding) will develop or not depends on the concentration of the sinks for vacancies in the reaction zone—the dislocations. After explosive welding, there are two transition zones with very different dislocation densities. An open question that arises after these analyses is if high detonation velocity, which results in higher deformation (and hence, higher dislocation densities), may suppress the nucleation of pores.

## 4. Conclusions

Detailed examination of the A1050/Ni201 explosively welded clads manufactured by significantly different experimental conditions and subjected to the additional annealing has been thoroughly studied. Independently of the colliding plates’ mutual localization, the value of the stand-off distance and the detonation velocity, the interfaces formed between the aluminum and nickel alloys were composed of the continuous melted layer. The chemical composition of the mixing zone consisting of (Al) + Al_3_Ni eutectic mixture, Al_3_Ni, Al_3_Ni_2_ and AlNi phases was always strongly inhomogeneous. Higher detonation velocity contributed to the creation of the Al_9_Ni_2_ metastable phase. In both examined cases, the inclusions of pure nickel within the mixing zone were also identified.

The various initial joining conditions had pronounced implications after a long time of annealing at 500 °C the clads, clearly shown in the current paper. Annealing at 500 °C resulted in creation of two layered phases, Al_3_Ni and Al_3_Ni_2_, at the examined interfaces. Both of the intermetallics existed only after 0.5 h of annealing in the case of clad obtained using lower detonation velocity, while after a longer annealing time, the presence of only Al_3_Ni_2_ phase at the interface was found. In the case of the second type of clad, the Al_3_Ni and Al_3_Ni_2_ phases were detected after each applied annealing time. It was also confirmed that during the annealing at 500 °C, the mobility of Al atoms is significantly faster than Ni, as it was evidenced by the pronounced Kirkendall effect manifested by porosity at A1050/Al_3_Ni_2_ and Al_3_Ni/Al_3_Ni_2_ interfaces and outward the curvature of the Al_3_Ni phase at the interface with A1050 alloy. Flat morphology of the A1050/Ni201 interface, faster consumption of Al_3_Ni, the crystallographic texture of the Al_3_Ni_2_ phase and accompanying appearance of an intense porosity at the Al1050/Al_3_Ni_2_ interface, all observed at lower detonation velocity, resulted in cracking of this clad after prolonged annealing followed by slow furnace cooling.

## Figures and Tables

**Table 1:** Chemical compositions of the initial plates.

**Alloy**	**Ni**		**Cu**	**Fe**	**Mn**	**C**	**Si**		**S**
**Ni 201**	99.0		0.25	0.40	0.35	0.02	0.35		0.01
**Alloy**	**Al**	**Fe**	**Si**	**Zn**	**Ti**	**Mg**	**Mn**	**Cu**	**Other**
A1050	99.50	0.40	0.25	0.07	0.05	0.05	0.05	0.05	0.03

**Table 2:** Parameters of the explosive welding process.

Samples Name	Flyer Plate	Base Plate	Thickness of Flyer Plate	Thickness of Base Plate	Detonation Velocity	Stand of Distance	Temp. of Heat Treatment	Time of Heat Treatment
E2000	A1050	Ni201	1 mm	1 mm	2000 m/s	2h	500 °C	0.5–168 h
E2800	Ni201	A1050	1 mm	1 mm	2800 m/s	1h	500 °C	0.5–168 h

**Table 3:** The chemical compositions obtained by SEM/EDS point analysis located in the regions marked with numbers 1–3 in Figure 3b,d.

Point	Average Content of Element in at. %		
	Al	Ni	Phase
1a	74.4 ± 1.5	25.6 ± 0.5	Al_3_Ni
1b	81.2 ± 1.6	18.8 ± 0.4	(Al) + Al_3_Ni
2	86.0 ± 1.7	14.0 ± 0.3	(Al) + Al_3_Ni
3	0.3 ± 0.3	99.7 ± 2.0	(Ni)

**Table 4:** The chemical compositions obtained by SEM/EDS point analysis located in the regions marked with numbers 1–4 in Figure 4d.

Point	Average Content of Element in at. %		
	Al	Ni	Phase
1	52.8 ± 1.15	47.2 ± 0.9	AlNi
2	71.9 ± 1.4	28.1 ± 0.6	Al_3_Ni
3	0.3 ± 0.3	99.7 ± 1.9	Ni
4	59.7 ± 1.2	40.3 ± 0.8	Al_3_Ni_2_

## Data Availability

The raw/processed data required to reproduce these findings cannot be shared at this time as the data also form part of an ongoing study.
